# Retraction: Comparative immunogenicity of HIV-1 gp160, gp140 and gp120 expressed by live attenuated newcastle disease virus vector

**DOI:** 10.1371/journal.pone.0244046

**Published:** 2020-12-14

**Authors:** The PLOS ONE Editors

After this article [1] was published, concerns were raised about results reported in Figures 2, 3, and 4.

Specifically:

- In Figure 2A, there appears to be a vertical discontinuity suggestive of possible image splicing after lane 3, and when levels are adjusted, the background appears different in lanes 1–3 versus lanes 4–5.
- In Figure 3, panels c and d appear to report overlapping fields of the same microscopy image: the lower right area of panel c appears to overlap with the lower left area of panel d. These panels represent the results of experiments using different infection conditions. This issue was confirmed by the University of Maryland following their investigation of this article.
- In Figure 4, there appear to be vertical discontinuities suggestive of possible image splicing after lanes 1, 2, 4, and 8.

The authors noted that the original data underlying the figures in question are no longer available, although they offered to address the issues via replication data. They further stated that the majority of data supporting other figures and files in this article are available as “soft copy” (computer files), and that the raw data including gel images, ELISA readings, and original laboratory notebooks are held by the University of Maryland.

The image concerns cannot be fully clarified in the absence of the original data, and so the *PLOS ONE* Editors retract this article.

SKS did not agree with retraction. SKK and PLC did not agree with retraction and stand by the article's findings, although PLC apologized for the issues with the article. The other authors either could not be reached or did not respond directly.
